# A novel AllGlo probe-quantitative PCR method for detecting single nucleotide polymorphism in CYP2C19 to evaluate the antiplatelet activity of clopidogrel

**DOI:** 10.1038/s41598-024-52540-3

**Published:** 2024-01-29

**Authors:** Hongwei Li, Yizhen Fang, Yongquan Chen, Yuning Lin, Zanxi Fang, Zhiyuan Lin, Huabin Xie, Zhongying Zhang

**Affiliations:** 1 Medical Laboratory Center, Xiamen Humanity Hospital, Fujian Medical University, No. 3777, Xianyue Road, Huli District, Xiamen, 361009 Fujian China; 2 Department of Laboratory Medicine, The Third Affiliated Hospital of Zhengzhou University, Zhengzhou, 450052 China; 3 Department of Clinical Laboratory, Xiamen Cardiovascular Hospital of Xiamen University, School of Medicine, Xiamen University, Xiamen, China; 4 Department of Medical Laboratory Center, Xiamen University Affiliated Zhongshan Hospital, Xiamen, 361004 China; 5 Department of Clinical Laboratory, Xiamen Hospital of Traditional Chinese Medicine, Xiamen, 361001 China; 6 Zhengzhou Key Laboratory for In Vitro Diagnosis of Hypertensive Disorders of Pregnancy, Zhengzhou, 450052 China; 7 Xiamen Key Laboratory of Precision Medicine for Cardiovascular Disease, Xiamen, 361009 China; 8 Xiamen Key Laboratory for Biomarkers and Translational Medicine, Xiamen, 361009 China

**Keywords:** Biological techniques, Genetics, Molecular biology, Biomarkers, Molecular medicine

## Abstract

CYP2C19 gene has multiple single nucleotide polymorphism (SNP), which is the major determinant for clopidogrel treatment responses. Therefore, CYP2C19 SNP detection is essential for predicting clopidogrel efficacy. Currently, there is still no quick and effective method for routine detection of common CYP2C19 SNPs in clinical laboratories, which is critically needed prior to clopidogrel treatment. AllGlo™ based quantitative PCR was used to develop a novel genotyping method for CYP2C19 SNP detection, termed CyPAllGlo. The performance of CyPAllGlo was compared with that of the commonly used fluorescence in situ hybridization (FISH) method, and the data was verified by DNA sequencing. CyPallGlo was used to identify CYP2C19 polymorphisms in 363 patients with coronary heart disease. The univariate analysis was used to access the antiplatelet efficacy of clopidogrel in patients. The associations between CYP2C19 polymorphisms and clopidogrel efficacy were analyzed. Using CyPAllGlo to detect CYP2C19*2 and CYP2C19*3 alleles was highly specific and fast. The detection limit was approximately 0.07 µg/µl and 0.7 µg/µl for CYP2C19*2 and CYP2C19*3, respectively. The consistency between FISH and CyPAllGlo were 98.07% for CYP2C19*2 and 99.17% for CYP2C19*3. DNA sequencing showed that the accuracy of CyPAllGlo was 100%. The analysis time for the whole CyPAllGlo procedure was approximately 60 min. Univariate analysis showed that the anticoagulation efficacy of clopidogrel was related to patient age, CYP2C19 genotype, metabolic phenotype, and LDL level. The logistic regression analysis showed that the genotype of CYP2C19 and metabolic phenotype was the two risk factors for clopidogrel antiplatelet ineffectiveness. This novel CyPAllGlo is a rapid and accurate method for detection of CYP2C19 SNP. The specificity and consistency of CyPAllGlo are comparable with that of widely used DNA sequencing. These findings provide valuable rapid method for predicting clopidogrel efficacy, which can be quickly translated to improve personalized precision medicine for coronary heart disease treatment.

## Introduction

Clopidogrel one of the two most used antiplatelet drugs, which reduces the risk of acute myocardial infarction and stroke and is a second-line preventive medication drug for atherosclerosis^1,2^. Clopidogrel is a prodrug that undergoes a two-step oxidation process in the human body^3,4^. It is converted to an inactive thiolactone metabolite first and then an active metabolite catalyzed by multiple cytochrome P450 (CYP) enzymes in the liver, which include CYP2C19^5,6^. CYP2C19 gene is highly polymorphic, which encodes multiple variants that vary in their catalytic activities. It has been found that CYP2C19 genotypes are associated with clopidogrel resistance, which underlies various anticoagulation activity of clopidogrel in populations with distinct genetic backgrounds^7–9^.

There is a high frequency to develop clopidogrel resistance in the Chinese population who is also more likely to develop thrombosis^10–12^. At present, different single nucleotide polymorphisms (SNPs) of CYP2C19 have been found, including CYP2C19*2, CYP2C19*3, CYP2C19*17, and others^13–16^. Based on the CYP2C19 SNP, the metabolism of clopidogrel can be divided into three types: extensive metabolizer (EM), intermediate metabolizer (IM) and poor metabolizer (PM). Both EM and IM are termed as normal metabolizers. Although CYP2C19*17 variant is associated with rapid drug metabolism, it is relatively rare in the Chinese population^17,18^. CYP2C19*2 (rs4244285, c.681G > A) and CYP2C19*3 (rs4986893, c.636G > A) are the two most common SNP alleles in Chinese, both of which have low catalytic activities. According to the 2013 Clinical Pharmacogenetics Implementation Consortium Guidelines, individuals who have homozygous CYP2C19*1 SNP (CYP2C19*1/*1) are EM. Individuals who carry one copy of CYP2C19*2 or CYP2C19*3, such as CYP2C19*1/2 or CYP2C19*1/3 SNP are IM. Individuals who carry homozygous CYP2C19*2/*2 or CYP2C19*3/*3 or heterozygous CYP2C19*2/*3 alleles are PM^19,20^, which occurs in about 14% of Chinese. In patients with PM, the production of active clopidogrel metabolites is insufficient due to decreased CYP2C19 enzymatic activity. Therefore, the PM patients are less responsive to clopidogrel and have high risk to develop thrombosis^21^.

Due to the role of CYP2C19 polymorphisms in anticoagulation therapy, a quick and cost-effective CYP2C19 genotyping method is urgently needed. To date, several genotyping methods have been developed to detect CYP2C19 SNPs. Among them, the PCR–restriction fragment length polymorphism (PCR–RFLP) analysis^22–25^, which is complicated and time-consuming. Several real time PCR-based techniques have also been developed to genotype CYP2C19, such as TaqMan probe^26,27^, high resolution melt (HRM)^28^, fluorescent resonance energy transfer (FRET)^29^, and tetra-primer amplification refractory mutation system-PCR (T-ARMS-PCR)^30^. However, the first four mentioned technologies are time consuming, labor intensive, complicate, and expensive. T-ARMS-PCR can only be used to detect a single known genetic variant each time and requires electrophoresis to demonstrate the results. In addition, the formation of primer dimers also complicates the interpretation of melting curves.

Herein we report that using the AllGlo technology, a simple and sensitive fluorescence-based real time quantitative PCR system, we developed a highly stable and sensitive method to identify CYP2C19 SNPs, designated CyPallGlo that included 2 PCR primers and 2 AllGlo probe. The method can be used simultaneously to identify CYP2C19*2 and CYP2C19*3 SNP in a single PCR reaction.

## Materials and methods

### Human subjects

A total of 363 CAD patients who undergo clopidogrel treatments were recruited from the Xiamen University affiliated Cardiovascular Hospital from July 2017 to June 2021. The recruitment was in compliance with the ethical policy of the hospital. The study was approval by the Ethics Committee of the Cardiovascular Hospital and followed the principles of the Declaration of Helsinki.

### Sample preparation

Genomic DNA was extracted from human peripheral blood (collected in EDTA antiplatelet tubes) using the Whole Blood Genomic DNA Extraction Kit (Tiangen, China) according to the manufacturer’s instructions. The concentration of DNA was measured with the Infinite 200 Pro NanoQuant (TECAN) kit. The isolated DNA samples were stored at − 80 °C until being used.

### PCR primers and AllGlo probes

The sequence of human CYP2C19 allele was downloaded from UCSC Genome Browser (http://genome.ucsc.edu). SNPs of CYP2C19 were obtained from the dbSNP database (http://www.ncbi.nlm.nih.gov). Comparisons with homologous nucleic acid sequences were made with the NCBI basic local alignment search tool (BLAST). The primers (PAGE purified) and probes (HPLC purified) for CYP2C19*2 (c.681G > A, rs4244285) and CYP2C19*3 (c.636G > A, rs4986893) were designed with Primer Premier 5.0. The oligonucleotide sequences of PCR products and probes for the SNPs of CYP2C19*2 and CYP2C19*3 (MAR-labeled and JUP-labeled) were shown in Supplementary Table 1.

### Cloning and analysis of quality control DNA templates

The PCR products of four SNP alleles (rs4244285, rs4986893, rs9934438, rs1057910, and rs3909184) were purified with the Universal DNA purification kit (Tiangen, China), cloned into the pMD18-T vector (Takara, Japan), and sequence verified. The sequences of rs4244285, rs4986893, rs9934438, rs1057910 and rs3909184 were shown in Supplementary Table 2.

### PCR amplifications

Dual-fluorescence PCR was carried out in 10 μl reaction mixture containing 1 μl gDNA (≈ 60 ng/μl), 5 μl AllGlo Probe qPCR Master Mix (1X, Yiyue, Shanghai), 0.2 μl primers (10 μM), 0.2 μl MAR-labeled probe (10 μM, Yiyue, Shanghai), and 0.2 μl JUP-labeled probe (10 μM, Yiyue, Shanghai) according to manufacturer’s instruction. The reference plasmids (≈ 70 ng/μl) for each SNP were used for quality control. For example, pMD18-T/681G to simulate 681G/G homozygous, pMD18-T/681A to simulate 681A/A homozygous, and 1:1 mixture of pMD18-T/681G and pMD18-T/681A to simulate 681G/A heterozygotes. The other four SNP references were used in the same way. A non-template control (NTC) was included in each assay. The optimized cycling condition for CYP2C19*2 and CYP2C19*3 were: 3 min at 95 °C; 40 cycles of 95 °C for 30 s, 60 °C for 34 s. The PCR products were separated by 2% agarose gel electrophoresis for specificity validation. In addition, the PCR products were purified for sequence verification.

### FISH analyses

The Cyp2C19 polymorphism detection kit (Jinan Guangyin Medical Technology company) was used to identify specific CYP2C19 alleles by the FISH method. The PCR condition for CYP2C19*2 and CYP2C19*3 was 50 cycles of 95 °C for 10 s, 60 °C for 40 s. The multichannel fluorescence quantitative analyzer (Fascan48S) was used for temperature control process and fluorometric analysis. The Cyp2C19 polymorphism detection kit contains specific double Z probes and a signal amplification probe. The double stranded PCR products were spread apart in 95 °C to yield single strand DNA for being hybridized with specific double Z probes in situ. The fluorescence emitted by the signal amplification probe and Z probes was then quantitated.

### Evaluation of anticoagulation efficacy and platelet count

Blood samples were collected for CAD patient after taking clopidogrel for six hours. The thromboela-stogram (TEG) was used to evaluate the anticoagulation efficacy. The Mindray BC6900 Automated Hematology Analyzer was used for platelet counting. Suppression of over 30% adenosine diphosphate (ADP)-induced coagulation is considered significant as described^31,32^.

### Genotyping and statistical analysis

Endpoint Genotyping by LightCycler 480 II was used to quantitate DNA according to the manufacturer's instruction. Cycle threshold (Ct) ≤ 34 was regarded as positive and Ct > 34 was regarded as negative. Non-normally distributed data was analyzed using Mann–Whitney U test. Normally distributed data was analyzed using the Student’s t-test. The Chi-square test was used to determine whether the differences were statistically significant. The Kappa consistency test was used to evaluate the consistency. Univariate analysis and logistic regression analysis were used to analyze the association between CYP2C19 polymorphisms and the antiplatelet efficacy of clopidogrel. Multiple factors were included to assess the association of drug efficacy and patient, including drug type, patient gender, age, CYP2C19 genotype, metabolic phenotype, myocardial infarction, hypertension, diabetes, smoking, preoperative/postoperative platelet count, and blood levels of triacylglycerol (TG), low density lipoprotein cholesterol (LDL), and Creatinine (CRE). Variables were assigned as follows in the logistic regression analysis: anti-platelet efficacy was divided into effective (assigned 1) and ineffective (assigned 0); binary class variable factors were assigned to 1 for yes and 2 for no; multiple class variables were assigned dummy variables with the variables to be included in the analysis were assigned 1, and other variables not included in the analysis were assigned. Statistical analysis were performed using SPSS 25.0 statistical software and GraphPad Prism version 5.01. *P* value < 0.05 was considered statistically significant.

### Ethics approval and consent to participate

The study followed the principles of the Declaration of Helsinki and was approved by the Ethics Committee of Cardiovascular Hospital of Xiamen University before the study was carried out. In this study, the remaining blood samples from the hospital diagnosis and treatment of the patients with coronary heart disease were used for detection and analysis, which would not cause any impact on the patients. Also, medical information of the patients was kept strictly confidential and the patients' information was de-identified in data analysis, ensuring no risk of information disclosure. Exemption of informed consent had been agreed by the Ethics Committee of Cardiovascular Hospital of Xiamen University.

## Results

### Development of CyPallGlo, an AllGlo based PCR for identifying CYP2C19*2 and CYP2C19*3 SNPs

**Figure 1:**
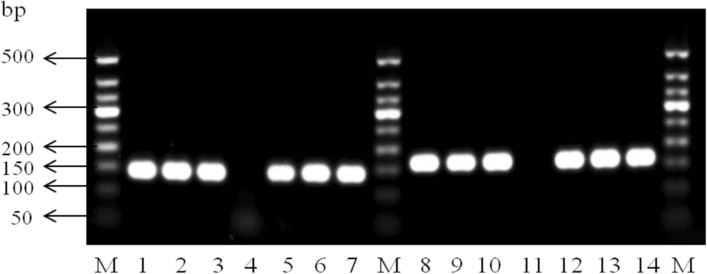
PCR products of the CYP2C19*2 and CYP2C19*3 alleles in genomic DNA and reference plasmid. CYP2C19*2 and CYP2C19*3 allele-positive samples (lanes 1, 5, 8, 12 are AA, lanes 2, 6, 9, 13 are GG, lanes 3, 7, 10, 14 are GA) showed amplification of all 3 genotypes’ products in genomic DNA (lanes 1–3, 8–10) and plasmid references (lane 5–7, 12–14), with products being about 147 bp (CYP2C19*2 alleles) and 168 bp (CYP2C19*3 alleles). The negative controls of CYP2C19*2 (lane 4) and CYP2C19*3 (lane 11) alleles had no non-specific products.

Approximately 60 ng of genomic DNA and 70 ng reference plasmid DNA were used for PCR amplification. The products were separated by 2% agarose gel electrophoresis for 40 min (Fig. 1). The results showed that all PCR products were uniform and high-yield, with an expected length between 100 and 200 bp, indicating that the PCR primers for the CyPallGlo analyses were could amplify the target DNA effectively.

To evaluate the accuracy of the method, a double-blind method was applied to detect CYP2C19*2 and CYP2C19*3 alleles in 40 reference plasmids (20 samples at a high concentration and 20 samples at a low concentration), each of which carried one of the two SNP alleles. The double-blind method showed that CYP2C19*2 and CYP2C19*3 were accurately detected by the AllGlo probe as expected, with Kappa = 1.00 for Kappa consistency test (Table 1).

**Table 1:** CyPallGlo detection of reference plasmid carrying CYP2C19*2 and CYP2C19*3 SNPs.

Number (concentration)	Genotyping result	Actual number/Experimental number	
		CYP2C19*2	CYP2C19*3
High concentration level	AA	6/6	6/6
	GG	7/7	7/7
	GA	7/7	7/7
Low concentration level	AA	6/6	6/6
	GG	8/8	8/8
	GA	6/6	6/6
	Kappa = 1.00		

**Figure 2:**
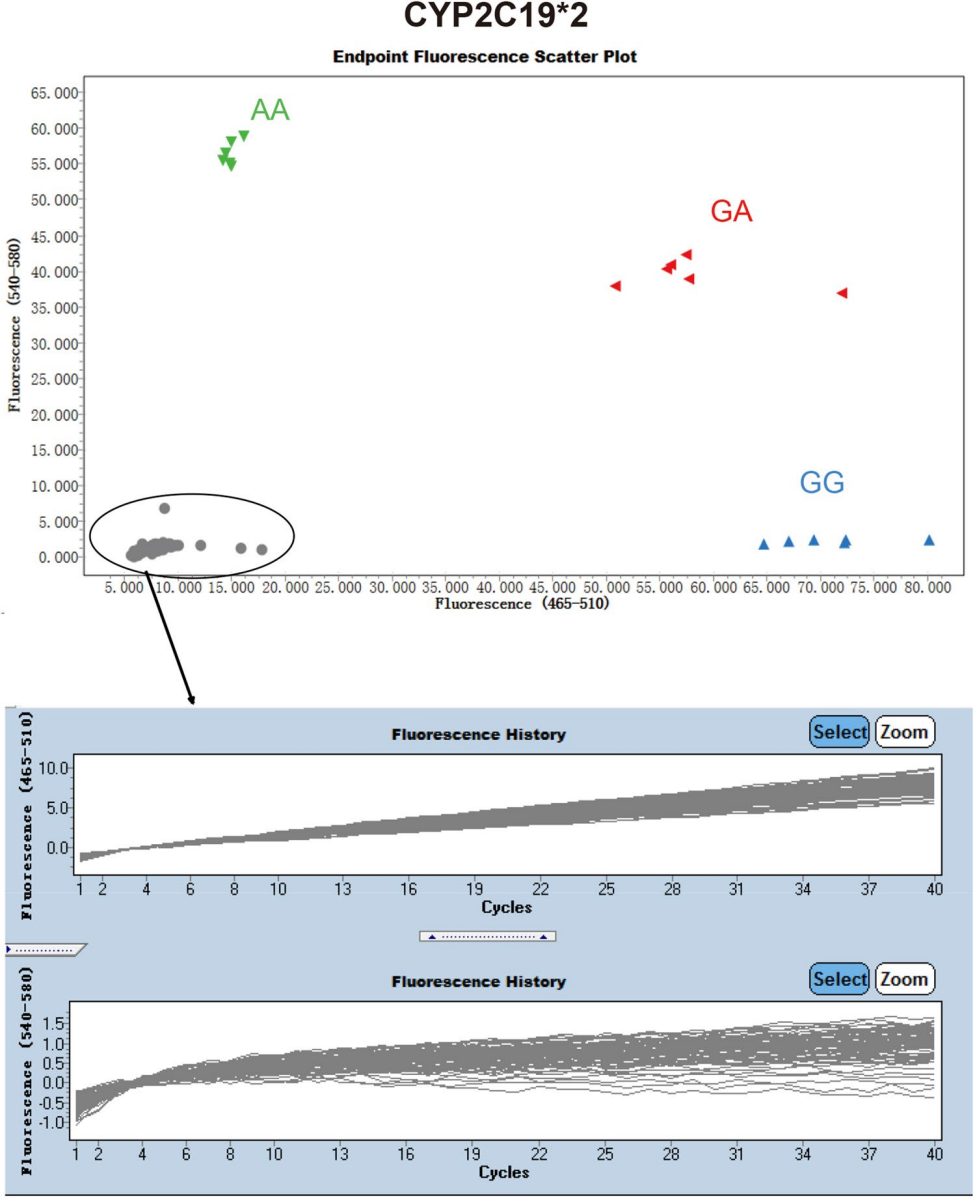
Specificity of CyPAllGlo in CYP2C19*2 detection. Three amplification curves of CYP2C19*2 alleles were distinguished from the quality control references with rs4244285, rs4986893, rs9934438, rs1057910 and rs3909184. No amplification curve was detected in the five quality control references using CyPAllGlo amplification system. The green, red, blue and gray dot clusters represented AA, GA, GG and no genotyping results respectively.

**Figure 3:**
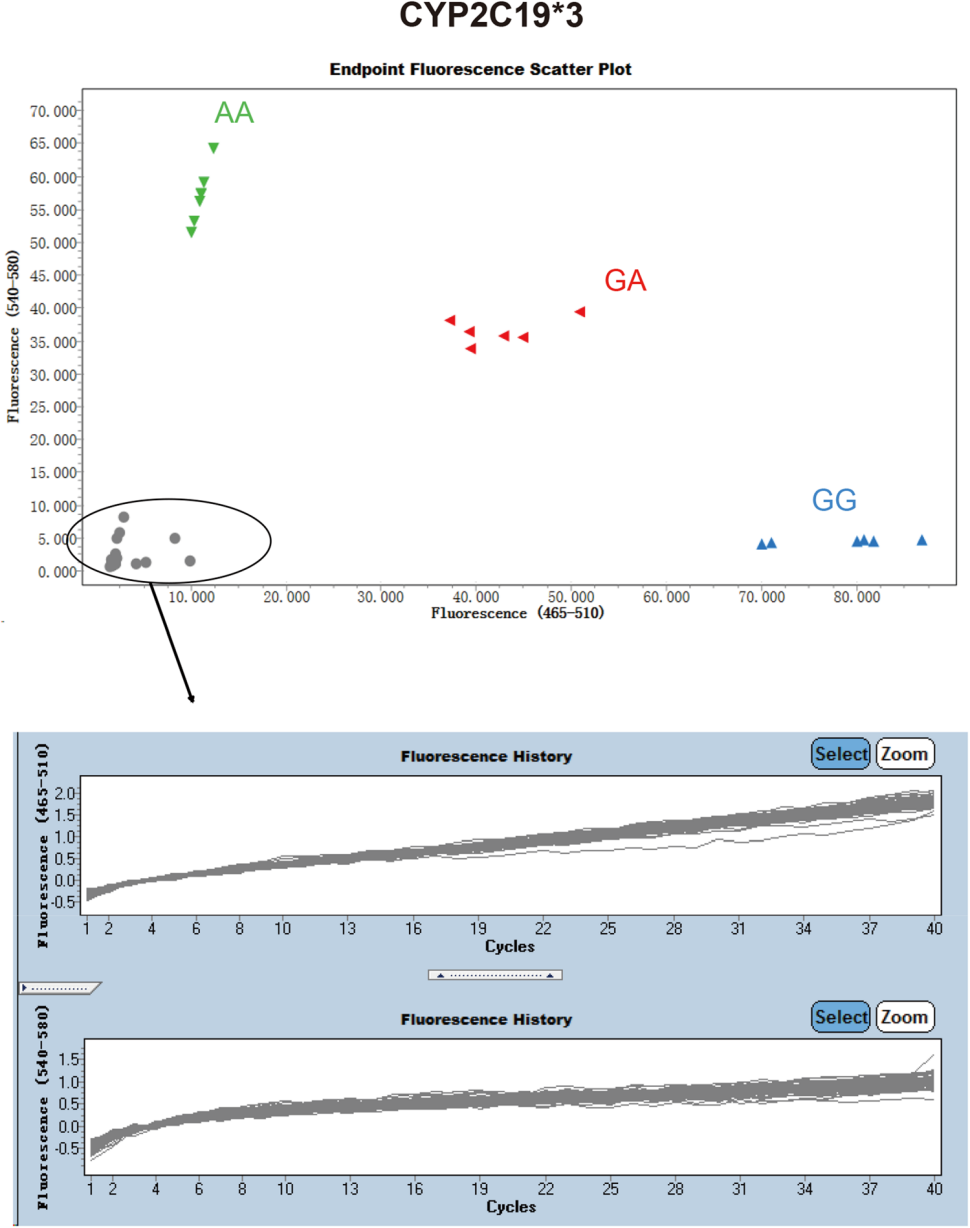
Specificity of CyPAllGlo in CYP2C19*3 detection. Three amplification curves of CYP2C19*3 alleles were distinguished from the quality control references with rs4244285, rs4986893, rs9934438, rs1057910 and rs3909184. No amplification curve was detected in the five quality control references using CyPAllGlo amplification system. The green, red, blue and gray dot clusters represented AA, GA, GG and no genotyping results respectively.

To determine whether CyPallGlo could the specifically identify the CYP2C19*2 and CYP2C19*3, the control references plasmids rs4244285, rs4986893, rs9934438, rs1057910, and rs3909184 were used for cross-reaction test. The data clearly showed that the CYP2C19*2 specific primers could only detect CYP2C19*2 SNP (Fig. 2) and that CYP2C19*3 specific primers could only detect CYP2C19*3 SNP (Fig. 3). No cross detection was observed.

**Figure 4:**
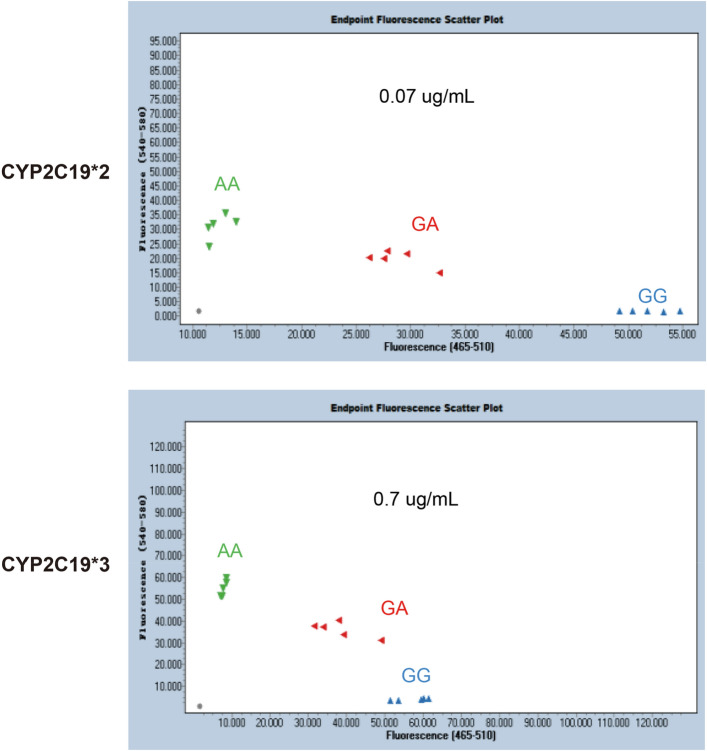
Sensitivity of CyPAllGlo in CYP2C19*2 (top section) and CYP2C19*3 detection (bottom section). Three CYP2C19*2 alleles were detected at the minimum detection limit of 10^4^ copies/μl, and three genotypes of CYP2C19*3 alleles were detected at the minimum detection limit of 10^5^ copies/μl. The green, red and blue point clusters represented AA, GA and GG, respectively. Negative control (NTC) was shown in gray dots.

To determine the sensitivity of the AllGlo PCR detection of CYP2C19*2 and CYP2C19*3, the reference plasmids carrying CYP2C19*2 and CYP2C19*3 SNP were tenfold serial diluted before being used for the analyses. All three genotypes were included, and the experiments were repeated 15 times. The results showed that as low as approximately 0.07 µg/ml of CYP2C19*2 and 0.7 µg/ml of CYP2C19*3 were detected with the AllGlo probe (Fig. 4), indicating that the detection limit for CYP2C19*2 allele was 10^4^ copies/μl, and that of CYP2C19*3 allele was 10^5^ copies/µl.

**Figure 5:**
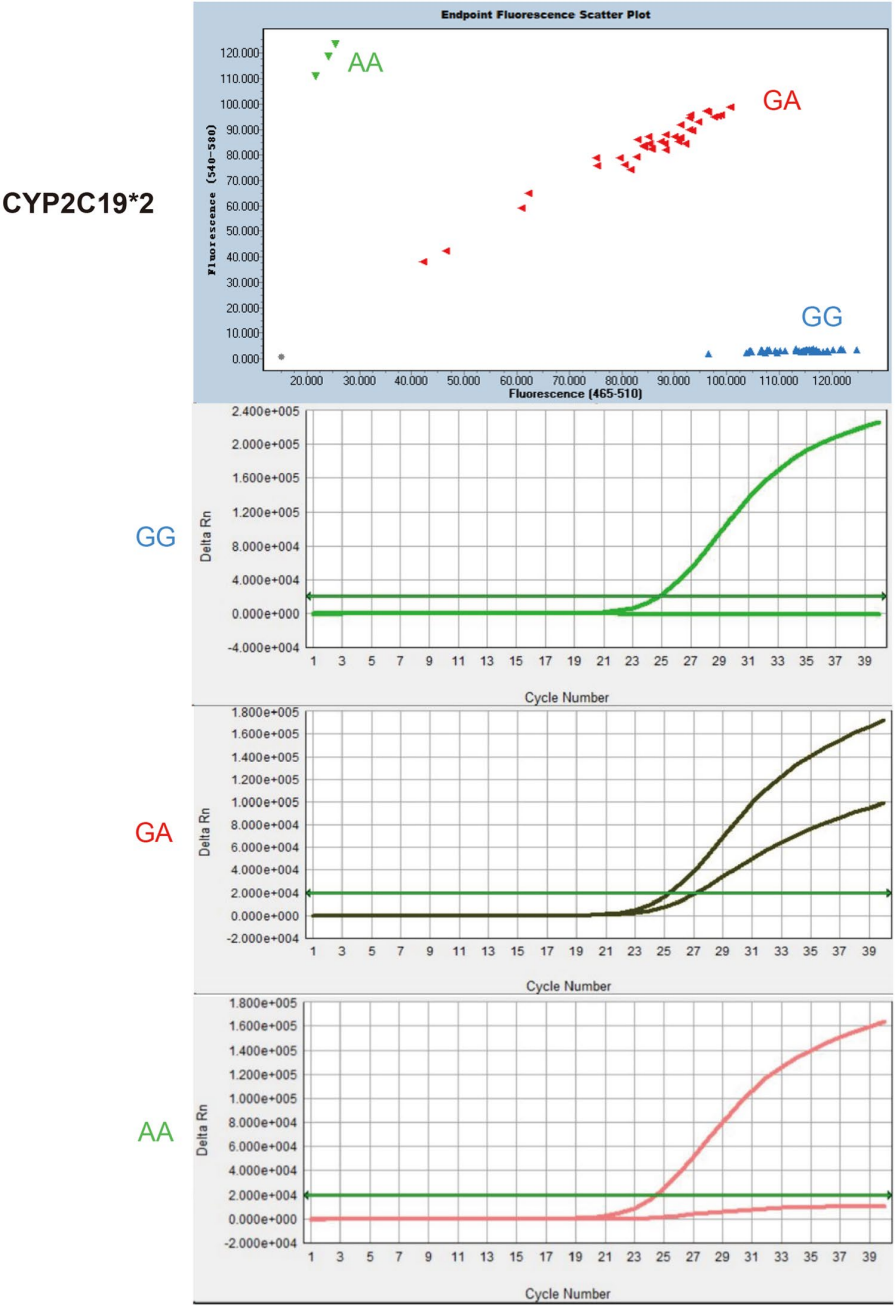
Accuracy of CyPAllGlo in CYP2C19*2 detection. The wild type of CYP2C19 (GG, blue dots) had only one corresponding amplification curve. CYP2C19*2 homozygous genotype (AA, green dots) and heterozygous genotype (GA, red dots) had one and two distinctive S-shaped specific amplification curve(s) within the same 34 Ct range, respectively.

**Figure 6:**
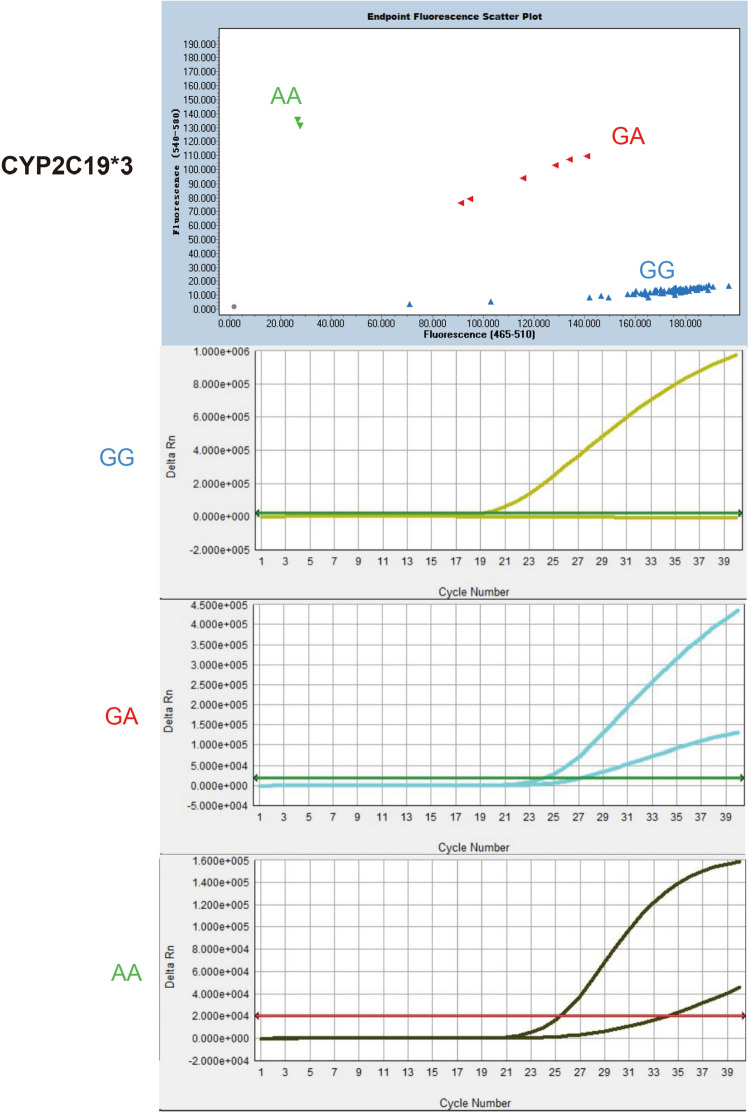
Accuracy of CyPAllGlo in CYP2C19*3 detection. The wild type of CYP2C19 (GG, blue dots) had only one corresponding amplification curve. CYP2C19*3 homozygous genotype (AA, green dots) and heterozygous genotype (GA, red dots) had one and two distinctive S-shaped specific amplification curve(s) within the same 34 Ct range, respectively.

To determine whether CyPallGlo could be used to detect CYP2C19 SNP correctly in human blood samples, a total of 363 genomic DNA samples were tested in Cobas Z 480 Analyzer (Roche) and ABI 7500 Real-time PCR System (Applied Biosystems) with CyPallGlo probes, respectively. All samples were randomly tested 4 times a day for 5 days. The results showed that all 363 clinical samples were genotyped correctly and equally well on the two instruments (Figs. 5 and 6), indicating that the CyPallGlo method could be used to identify CYP2C19 SNP in clinical samples.

### CyPAllGlo identifies Cyp2C19 SNPs more accurately than the traditional FISH and can be used to predict the efficacy of anti-platelet treatments

To assess the clinical value of CyPAllGlo in detecting CYP2C19 SNPs, we compared the accuracy of CyPAllGlo and the traditional FISH in detection of CYP2C19 SNPs in the pool of 363 human blood samples. The results showed that the two detection methods shared 98.07% consistence in detecting CYP2C19*2 (356/363) and 99.17% in detecting CYP2C19*3 (360/363), respectively. Among the 10 samples with inconsistent results between the two detection methods, DNA sequence confirmed that the CyPAllGlo data were all correct, with Kappa = 1.00 for Kappa consistency test. We then randomly selected 50 samples from the consistent group for DNA sequencing. The results showed that all the results were accurate (Table 2). Together, the results indicate that CyPAllGlo was significantly more accurate than FISH in detecting CYP2C19 SNPs.

**Table 2:** Comparison of CyPAllGlo and DNA sequencing detection of CYP2C19*2 and CYP2C19*3 in 50 randomly selected clinical samples.

Allele	Genotyping result	Sequencing method
		Sanger sequencing/AllGlo probe based sequencing
CYP2C19*2	AA	4/4
	GG	21/21
	GA	25/25
CYP2C19*3	AA	2/2
	GG	41/41
	GA	7/7
	Kappa = 1.00	

To determine whether CyPAllGlo results could be used to predict the efficacy of anti-platelet treatments, statistical analyses was employed to assess the association of drug efficacy and patient health information, including gender, age, CYP2C19 SNPs, metabolic phenotype, myocardial infarction, hypertension, diabetes, smoking history, preoperative/ postoperative platelet count, and blood levels of TG, LDL, and Creatinine. Excluding the data incomplete cases, a total of 288 patients were included in the analysis, which included 210 clopidogrel-treated cases and 78 ticagrelor-treated cases. An inhibition rate ≥ 30% was considered effective, and an inhibition rate < 30% was considered ineffective.

Univariate analysis showed that both clopidogrel and ticagrelor had good antiplatelet activity. (92.38% and 92.31%, respectively). In the clopidogrel group of 210 patients, univariate analysis showed that the antiplatelet activity was related to patient age, CYP2C19 genotype, metabolic phenotype, and LDL level (Table 3). Logistic regression analysis showed that the genotype of poor metabolizer (PM) were risk factors for clopidogrel ineffectiveness (Table 4). However, univariate analysis showed that the antiplatelet activity of ticagrelor was not related to any of these factors in 78 patients treated with ticagrelor (Table 5). The results indicate that CyPAllGlo can be used to screen patients who are responsive to clopidogrel treatment and that CYP2C19 SNP information can be used to guide the selection of appropriate antiplatelet drugs and their dosage in precision medicine.

**Table 3:** Analysis of influencing factors for clopidogrel resistance in 210 CAD patients.

Factors	Level	Effective	Not effective	95% CI	Statistics	*P* value
Sex				0.675–6.320	*χ*^*2*^ = 1.672	0.196
	Male	159	11			
	Female	35	5			
Genotype				2.326–1328.565	*χ*^*2*^ = 15.363	0.009*
	*1/*1	74	4			
	*1/*2	79	5			
	*1/*3	20	1			
	*2/*2	14	2			
	*2/*3	6	3			
	*3/*3	1	1			
Metabolic phenotype				3.824–128.623	*χ*^*2*^ = 13.873	0.001*
	EM	74	5			
	IM	99	5			
	PM	21	6			
Myocardial infarction				0.147–1.384	*χ*^*2*^ = 2.022	0.155
	Yes	33	5			
	No	161	11			
Hypertension				0.292–2.396	*χ*^*2*^ = 0.110	0.740
	Yes	113	10			
	No	81	6			
Diabetes				0.469–4.871	*χ*^*2*^ = 0.485	0.486
	Yes	65	4			
	No	129	12			
Smoking				0.388–3.304	*χ*^*2*^ = 0.024	0.876
	Yes	113	9			
	No	81	7			
Age (years old)		64.99 ± 12.32	70.75 ± 8.36	1.198–10.623	*t* = 2.468	0.023*
Preoperative platelet count (10^9^/L)		219.76 ± 51.76	223.44 ± 58.07	− 23.256–30.605	*t* = 0.238	0.815
Postoperative plateletcount (10^9^/L)		210.38 ± 51.40	220.69 ± 73.10	− 17.192–37.804	*t* = 0.536	0.599
TG (mmol/l)		1.74 (1.16, 2.65)	1.83 (1.16, 2.34)	− 0.349–1.130	*H* = 0.686	0.502
LDL (mmol/l)		2.44 (1.88, 3.11)	2.20 (1.65, 2.59)	1.042–2.158	*H* = 3.047	0.033*
CRE (mmol/l)		80.40 (68.48, 94.98)	78.00 (78.50, 87.43)	− 13.770–11.458	*H* = 0.208	0.838

95% CI refers to the 95% confidence interval for the statistic *X*^*2*^, *t* or *H*. Through Kolmogorov–Smirnov's normal test of continuous variables, the preoperative platelet count, postoperative platelet count and age were normally distributed, while the levels of TG, LDL-C and creatinine did not conform to normal distribution, so the preoperative platelet count, postoperative platelet count and age were represented by (mean ± standard deviation), and TG, LDL and CRE were represented by quartile P50 (P25, P75).

*95% CI* 95% confidence interval, *PM* poor metabotype, *IM* intermediate metabotype, *EM* extensive metabotype, *TG* triglycerides, *LDL* low density lipoprotein cholesterol, *CRE* creatinine; *X*^*2*^ Chi-square test statistic value, *t* t test statistic value, *H* Wilcoxon rank sum test statistic value.

**Table 4:** Logistic regression analysis of influencing factors for clopidogrel resistance in 210 CAD patients.

Parameter	B value	Standard Error	Wald	Significance	Exp (B)	95% Confidence Interval
Sex						
Male	0.692	0.681	1.032	0.310	1.997	0.526–7.592
Genotype						
*3/*3	3.877	2.036	3.626	0.027	48.300	1.043–7613.152
*2/*3	3.740	1.972	3.595	0.018	42.079	1.881–9263.744
*2/*2	4.535	2.282	3.950	0.023	93.251	1.065–8168.621
*1/*3	2.365	2.124	1.526	0.267	9.768	0.175–624.265
*1/*2	2.305	2.044	1.272	0.259	10.021	0.183–550.192
Metabolic phenotype						
PM	− 2.553	0.800	2.477	0.032	35.575	1.030–2.761
IM	0.066	0.630	0.011	0.917	1.068	0.311–3.670
Myocardial infarction						
Yes	− 0.794	0.619	1.645	0.200	0.452	0.134–1.521
Hypertension						
Yes	− 0.190	0.625	0.093	0.761	0.827	0.243–2.816
Diabetes						
Yes	0.571	0.642	0.793	0.373	1.771	0.503–6.230
Smoking						
Yes	− 0.548	0.651	0.709	0.400	0.578	0.161–2.070
Age	− 0.044	0.028	2.495	0.114	0.957	0.906–1.011
Preoperative platelet count	0.013	0.011	1.593	0.207	1.014	0.993–1.035
Postoperative platelet count	− 0.013	0.009	1.970	0.160	0.987	0.969–1.005
TG	0.053	0.218	0.059	0.808	1.054	0.688–1.615
LDL	0.465	0.341	1.860	0.173	1.592	0.816–3.105
CRE	0.006	0.013	0.192	0.661	1.006	0.980–1.032

*PM* poor metabotype, *IM* intermediate metabotype, *EM* extensive metabotype, *TG* triglycerides, *LDL* low density lipoprotein cholesterol, *CRE* creatinine, qualitative parameter reference, female, *1/*1, EM, no myocardial infarction, no hypertension, no diabetes, no smoking.

**Table 5:** Analysis of influencing factors for ticagrelor resistance in 78 CAD patients.

Factors	Level	Effective	Not effective	95% CI	Statistics	*P* value
Sex				0.220–23.220	*χ*^*2*^ = 0.471	0.493
	Male	66	5			
	Female	6	1			
Genotype				0.326–1108.635	*χ*^*2*^ = 1.112	0.892
	*1/*1	19	2			
	*1/*2	37	3			
	*1/*3	7	1			
	*2/*2	5	0			
	*2/*3	4	0			
	*3/*3	0	0			
Metabolic phenotype				0.289–166.243	*χ*^*2*^ = 0.877	0.645
	EM	19	2			
	IM	44	4			
	PM	9	0			
Myocardial infarction				0.127–3.576	*χ*^*2*^ = 0.216	0.642
	Yes	29	3			
	No	43	3			
Hypertension				0.039–3.194	*χ*^*2*^ = 0.925	0.336
	Yes	46	5			
	No	26	1			
Diabetes				0.106–3.006	*χ*^*2*^ = 0.457	0.499
	Yes	26	3			
	No	46	3			
Smoking				0.037–3.007	*χ*^*2*^ = 1.045	0.307
	Yes	45	5			
	No	27	1			
Age (years old)		58.67 ± 11.13	64.83 ± 15.44	− 3.713–16.046	*t* = 0.877	0.418
Preoperative platelet count (10^9^/L)		225.11 ± 58.84	255.67 ± 51.31	− 19.422–80.533	*t* = 1.274	0.250
Postoperative platelet count (10^9^/L)		214.42 ± 67.36	231.67 ± 46.23	− 39.317–73.817	*t* = 0.778	0.463
TG (mmol/l)		1.50 (1.10, 2.23)	1.29 (0.91,2.33)	− 1.391–1.682	*H* = 0.917	0.390
LDL (mmol/l)		2.57 (1.88, 3.11)	2.42 (1.45, 3.22)	− 0.963–1.567	*H* = 0.478	0.651
CRE (mmol/l)		90.45 (73.00, 107.13)	84.65 (70.93, 129.80)	− 92.910–84.071	*H* = 0.174	0.865

95% CI refers to the 95% confidence interval for the statistic *X*^*2*^, *t* or *H*. Through Kolmogorov–Smirnov's normal test of continuous variables, the preoperative platelet count, postoperative platelet count and age were normally distributed, while the levels of TG, LDL-C and creatinine did not conform to normal distribution, so the preoperative platelet count, postoperative platelet count and age were represented by (mean ± standard deviation), and TG, LDL and CRE were represented by quartile P50 (P25, P75).

*95% CI* 95% confidence interval, *PM* poor metabotype, *IM* intermediate metabotype, *EM* extensive metabotype, *TG* triglycerides, *LDL* low density lipoprotein cholesterol, *CRE* creatinine, *X*^*2*^ Chi-square test statistic value, *t* t test statistic value, *H* Wilcoxon rank sum test statistic value.

## Discussion

CYP2C19 is a cytochrome C family member that plays important roles in drug metabolism. The gene encoding CYP2C19 is highly polymorphic, and each variant has different activities in drug metabolism. CYP2C19 activates the antiplatelet drug clopidogrel. The polymorphisms in the CYP2C19 gene are known to alter the outcome for patients taking clopidogrel for cardiovascular disease treatment. Therefore, detection of CYP2C19 genotype is critical for selecting patients for antiplatelet treatments. However, current methods of analyzing CYP2C19 SNPs are tedious, expensive, and sometimes not reliable. Herein, we report to develop CyPAllGlo, a novel CYP2C19 SNP method based on the AllGlo technology, which is highly sensitive, relatively simple, and cost effective, which is more reliable than commonly used methods in identifying CYP2C19 SNPs.

Current methods of analyzing whole blood genomic DNA include PCR–RFLP, HRM, ARMS^30^, FISH^33^, TaqMan probe, and DNA sequencing^34^. Among them, PCR–RFLP is a common method for detecting genetic variations, including CYP2C19 SNPs. Although it is relatively inexpensive, it is tedious, time-consuming, and only suitable for some SNPs. In addition, its sensitivity and specificity are affected by DNA quality and quantity. HRM detects DNA sequence variations based on monitoring changes in the melting temperature of double-stranded DNA during PCR amplification. It is relatively fast, cost-effective, and can detect multiple SNPs simultaneously. However, it can not be able to detect new SNPs or variations that result in small changes in melting temperature. ARMS is a PCR based method and one of the most used in clinical practice. It uses allele-specific primers to amplify specific DNA fragments for SNP detections. It is relatively simple and inexpensive, but the sensitivity and fidelity are low. TaqMan probe is a real-time PCR method that uses fluorescent probes to detect the presence of specific DNA sequences. It is highly sensitive, specific, and can detect multiple SNPs simultaneously, which is currently the most widely used and commercially mature method. TaqMan probes are single oligonucleotides with a fluorophore and quencher, while AllGlo probes consist of two separate oligonucleotides carrying the fluorophore and quencher. This makes it possible for the AllGlo method to have a stronger signal and lower background, increasing detection sensitivity. Loop-mediated isothermal amplification (LAMP) is an innovative technique that is being developed for CYP2C19 genotyping. However, the application of LAMP is restricted by its binary amplification results, inability to amplify longer fragments, susceptibility to contamination, and challenges in product recovery and detection. In China, where fluorescence quantitative PCR instruments are widely used in clinical testing laboratories, the adoption rate of LAMP devices is not high. Therefore, the advantage of the CyPallGlo method lies in its compatibility with existing infrastructure^35–38^.

Unlike TaqMan probes that carry both fluorophore and quencher, AllGlo probes consist of two oligonucleotides. Each AllGlo probe carries the fluorophore or quencher separately, which makes it possible for the AllGlo probe-based method to generate strong signals with low background, and therefore, is highly sensitive. The signals are quantitative, which allows assessment of SNP abundance. In addition, flexible choice of AllGlo probes^39,40^ offers options to assure highly specific binding to target sequences, as well as avoiding issues with nonspecific amplification and probe dimmers, which yield false-positive results. Furthermore, the AllGlo probes can be used to amplify long DNA fragments, which makes it suitable for SNP analysis in large genomic regions.

The newly developed CyPAllGlo is an accurate, reproducible, and time-saving method for genotyping Cyp2C19 in blood samples. It has 100% consistent with DNA sequencing results. No difference was found with instrument from different vendors (Cobas Z 480 Analyzer and ABI 7500 Real-time PCR System). Cross-reaction experiments with DNA fragments carrying rs4244285, rs4986893, rs9934438, rs1057910, and rs3909184 alleles showed no false positive results. The results indicate that CyPAllGlo is highly specific for genotyping CYP2C19*2 and CYP2C19*3. In addition, CyPAllGlo is highly sensitive, it detected CYP2C19*2 and CYP2C19*3 at the level of 0.07 µg/µl and 0.7 µg/µl, respectively. Furthermore, CyPAllGlo provides a rapid platform for detecting CYP2C19*2 and CYP2C19*3 SNPs. The total analysis time is about 60 min from the input of genomic DNA sample to the output of data. The results are intuitive and easy to interpret. Since patients with PM capacity of clopidogrel should consider using alternative medication or dose adjustment. Our study confirmed that the CyPAllGlo was an accurate and rapid detection method for CYP2C19 SNP detection, which can be used to screen patients prior to clopidogrel treatment. Furthermore, our results showed that CyPAllGlo meets the clinical requirements for genotyping CYP2C19*2 and CYP2C19*3 SNPs in peripheral blood samples.

## Conclusions

CyPAllGlo is a novel and efficient method for identifying CYP2C19 SNPs associated with clopidogrel efficacy. It demonstrates high accuracy, rapidity, and consistency when compared to the commonly used FISH method and DNA sequencing. Statistical analysis revealed that specific CYP2C19 SNP and metabolic phenotypes were significant risk factors for clopidogrel ineffectiveness. The findings are of translational value in precision medicine for selecting patients with coronary diseases who are suitable for clopidogrel treatments.

### Supplementary Information

Supplementary Table 1.

Supplementary Table 2.

## Data Availability

The datasets used and/or analyzed during the current study are available from the corresponding author on reasonable request.

## Abbreviations

CYP2C19: Cytochrome P450 2C19
PCR–RFLP: PCR–restriction fragment length polymorphism
HRM: High resolution melt
FRET: Fluorescent resonance energy transfer
CAD: Coronary heart disease
SNP: Single nucleotide polymorphism
TG: Triacylglycerol
LDL: Low density lipoprotein cholesterol
CRE: Creatinine
