# Supplementary material for: A novel AllGlo probe-quantitative PCR method for detecting single nucleotide polymorphism in CYP2C19 to evaluate the antiplatelet activity of clopidogrel
Source: Sci Rep. 2024 Jan 29;14:2358. doi: 10.1038/s41598-024-52540-3 (PMC10825217; doi:10.1038/s41598-024-52540-3)
Supplement: Supplementary file 1 — Supplementary Table 1. [file 41598_2024_52540_MOESM1_ESM.doc]

**Supplementary Table 1 The primers, probes and estimated product sizes of CYP2C19*2 and CYP2C19*3**

| Allele | Oligonucleotide Sequences(5’-3’) | |
| --- | --- | --- |
| CYP2C19*2 | Forward primer | TGCAATAATTTTCCCACTATCATTG |
|  | Reverse primer | AATAAAGTCCCGAGGGTTGTTG |
|  | CYP2C19*2-G | MAR-TATTTCCCGGGAACC-MAR |
|  | CYP2C19*2-A | JUP-TTATTTCCCAGGAACC-JUP |
|  | Product Size (bp) | 147 |
| CYP2C19*3 | Forward primer | GATCAGCAATTTCTTAACTTGATGGA |
|  | Reverse primer | AAAATGTACTTCAGGGCTTGGTCA |
|  | CYP2C19*3-G | MAR-CCCCCTGGATCCAG-MAR |
|  | CYP2C19*3-A | JUP-CCCCCTGAATCCAG-JUP |
|  | Product Size (bp) | 168 |
