# Supplementary material for: A novel AllGlo probe-quantitative PCR method for detecting single nucleotide polymorphism in CYP2C19 to evaluate the antiplatelet activity of clopidogrel
Source: Sci Rep. 2024 Jan 29;14:2358. doi: 10.1038/s41598-024-52540-3 (PMC10825217; doi:10.1038/s41598-024-52540-3)
Supplement: Supplementary file 2 — Supplementary Table 2. [file 41598_2024_52540_MOESM2_ESM.doc]

**Supplementary Table 2 The partial sequences of rs4244285, rs4986893, rs9934438, rs1057910 and rs3909184**

| SNP | Base sequence |
| --- | --- |
| rs4244285-A | TGCAATAATTTTCCCACTATCATTGATTATTTCCC**A**GGAACCCATAACAAATTACTTAAAAACCTTGCTTTTATGGAAAGTGATATTTTGGAGAAAGTAAAAGAACACCAAGAATCGATGGACATCAACAACCCTCGGGACTTTATT |
| rs4244285-G | TGCAATAATTTTCCCACTATCATTGATTATTTCCC**G**GGAACCCATAACAAATTACTTAAAAACCTTGCTTTTATGGAAAGTGATATTTTGGAGAAAGTAAAAGAACACCAAGAATCGATGGACATCAACAACCCTCGGGACTTTATT |
| rs4986893-A | GATCAGCAATTTCTTAACTTGATGGAAAAATTGAATGAAAACATCAGGATTGTAAGCACCCCCTG**A**ATCCAGGTAAGGCCAAGTTTTTTGCTTCCTGAGAAACCACTTACAGTCTTTTTTTCTGGGAAATCCAAAATTCTATATTGACCAAGCCCTGAAGTACATTTT |
| rs4986893-G | GATCAGCAATTTCTTAACTTGATGGAAAAATTGAATGAAAACATCAGGATTGTAAGCACCCCCTG**G**ATCCAGGTAAGGCCAAGTTTTTTGCTTCCTGAGAAACCACTTACAGTCTTTTTTTCTGGGAAATCCAAAATTCTATATTGACCAAGCCCTGAAGTACATTTT |
| rs9934438-G | AAAGGTGATTTCCAAGAAGCCACCTGGGCTATCCTCTGTTCCCCGACCTCCCATCCTAGTCCAAG**G**GTCGATGATCTCCTGGCACCGGGCACCTTTGGCCACGTCAGGATTCCATGTCACTG |
| rs9934438-A | AAAGGTGATTTCCAAGAAGCCACCTGGGCTATCCTCTGTTCCCCGACCTCCCATCCTAGTCCAAG**A**GTCGATGATCTCCTGGCACCGGGCACCTTTGGCCACGTCAGGATTCCATGTCACTG |
| rs1057910-A | AGCCACATGCCCTACACAGATGCTGTGGTGCACGAGGTCCAGAGATAC**A**TTGACCTTCTCCCCACCAGCCTGCCCCATGCAGTGACCTGTGACATTAAATTCAGAAACTATCTCATTCCCAAGGTAAGTTTGTTTCTC |
| rs1057910-C | AGCCACATGCCCTACACAGATGCTGTGGTGCACGAGGTCCAGAGATAC**C**TTGACCTTCTCCCCACCAGCCTGCCCCATGCAGTGACCTGTGACATTAAATTCAGAAACTATCTCATTCCCAAGGTAAGTTTGTTTCTC |
| rs3909184-G | ATGTGCTCAGTGCCCTCAAGGTATTTATACTCTAGAAGGGGGCACA**G**GTGGGAAAAATAGATTAAACGGGGGCTCATCAGAG |
| rs3909184-C | ATGTGCTCAGTGCCCTCAAGGTATTTATACTCTAGAAGGGGGCACA**C**GTGGGAAAAATAGATTAAACGGGGGCTCATCAGAG |
